# Post-comatose patients with minimal consciousness tend to preserve reading comprehension skills but neglect syntax and spelling

**DOI:** 10.1038/s41598-019-56443-6

**Published:** 2019-12-27

**Authors:** Agnieszka Kwiatkowska, Michał Lech, Piotr Odya, Andrzej Czyżewski

**Affiliations:** Department of Multimedia Systems, Faculty of Electronics, Telecommunication and Informatics, Gdansk University of Technology, Gdańsk, Poland

**Keywords:** Brain injuries, Biomedical engineering

## Abstract

Modern eye tracking technology provides a means for communication with patients suffering from disorders of consciousness (DoC) or remaining in locked-in-state. However, being able to use an eye tracker for controlling text-based contents by such patients requires preserved reading ability in the first place. To our knowledge, this aspect, although of great social importance, so far has seemed to be neglected. In the paper, we presented the possibility of using an eye-tracking technology for assessing reading comprehension skills in post-comatose patients with minimal consciousness. We prepared various syllable-, word- and sentence-based tasks, controlled by gaze, used for assessing the reading comprehension skills. The obtained results showed that people with minimal consciousness preserved the reading comprehension skills, in most cases to a high extent, but had difficulties with recognizing errors in the written text. The ability to maintain attention during performing the tasks was in statistically significant correlation with motivation, and that one was in a statistically significant correlation with the reading ability. The results indicate that post-comatose patients with minimal consciousness can read words and sentences, hence some useful hints may be provided for the development of gaze tracking-based human-computer interfaces for these people.

## Introduction

Brain injuries can be divided into two main categories: non-traumatic brain injuries (Non-TBI) and traumatic brain injuries (TBI). Regardless of their aetiology, they have some typical consequences and they usually influence injured people’s lives in many aspects. People after brain injury suffer from memory difficulties, reduced language and communication skills^1^, and deficits in vision and hearing^2–4^. They may have problems with attention and concentration^5^, and difficulties in social functioning^6^. Disorders of consciousness are common in severe brain injuries^7,8^. Brain death is the most severe consequence of brain injury. Although the results of a recent study^9^ show that it is possible to restore microcirculation and molecular and cellular functions of the pig brain up to four hours post-mortem, in most countries a person is considered to be legally dead when brain activity ceases (although criteria for defining brain death vary across countries^10^). Comatose state, although visually similar at bedside to the brain death, might be sometimes followed by unresponsive wakefulness syndrome (UWS), minimally consciousness state (MCS), and emerging from the minimally consciousness state (EMCS). Assessment of brain injury severity is crucial for further rehabilitation of patients. The most common method of the assessment in acute phase is the Glasgow Coma Scale (GCS). However, the GCS does not test for eye movements^11^. Visual fixation and pursuit were recently shown to be the most frequent signs of consciousness in a sample of 282 MCS patients (i.e., it was present in more than 50% of them)^12^. These visual abilities may thus be considered as determinant in this population of patients.

As we showed previously^13^, tracking eye movements using a Human-Computer Interaction (HCI) system provided a unique possibility of quantifying on a numeric scale the ability of patients to perform various computer tasks. The tasks were aimed at assessing speech and reading comprehension at a basic level, and at assessing the ability to recognize digits and objects in images. The developed HCI was proposed as a tool complementary to the clinical scales. Instead of classifying definite states of patients, our HCI ranked their scores based on the odds ratios from the Fisher’s exact test to provide more precise information and thus improve identification of the actual state of consciousness. There was a basis to believe that the patient was in MCS (or with LIS) only if the distribution of answers given in the tasks was significantly different from the random distribution (p < 0.05, one-tailed Fisher’s exact test). However, performing those of the tasks which were word- or sentence-based, required the preserved reading ability which was not quantified in the scope of that previous research. Here, we present the results of research in which similar HCI technology was utilized for assessing the reading ability itself. Various reading comprehension skills were taken into account. Preliminary research results based on data collected from 14 patients had been previously reported^14^. There were two main findings of that research. The first finding was that the patients who were originally, and incorrectly, diagnosed with UWS were identified with MCS or with LIS. The second finding, related to the reading comprehension skills, was that 13 patients preserved the ability to read sentences and 12 patients preserved the ability to read single words. However, 5 patients were not able to identify syllables in words and 6 patients were not able to indicate the incorrectly placed letters. The motivation to perform the tasks and the ability to maintain attention were not examined in that study.

The ability to understand oral speech is one of the key factors that facilitate the rehabilitation process of DoC patients. It is also used for assessing patients’ levels of consciousness. Various modern behavioural scales (e.g. CSR-R) consist of a set of tests in which patients are asked to follow therapist’s instructions. Recently, a new, more sophisticated test, called CAVE (Cognitive Assessment by Visual Election) has been proposed^15^. The test also includes subtests related to comprehension of written words in MCS patients and patients emerging from the MCS (EMCS). Two stimuli (e.g., written letters or words) are presented on the left and on the right side of the patient. In a recent case series^16^, visual pursuit capacity was initially assessed with the CRS-R (i.e., without any eye-tracking setting) in 5 MCS or EMCS patients, and one of these patients also showed residual letter and word reading abilities using the CAVE (i.e., 9 or 10/10). These results were associated with absence of hypometabolism and grey matter hypotrophy in left-sided occipital lobule and language-related areas. Comprehension of sentences as well as syntactic and spelling skills were not assessed in that study. Observations were made by Gajardo-Vidal *et al*.^17^, according to which, damages in the left hemisphere, which is typically dominant in language processing, usually cause more serious disturbance in speech comprehension than the damages in the right hemisphere. Both mentioned studies have used functional neuroimaging techniques to assess differences in the excitation of various brain regions. This approach seems to be growing in popularity. Nigri *et al*.^18^ have used fMRI (and PET) to compare lexical processing in DoC patients. In general, patients’ brain responses to heard and seen words (and nonwords) were weaker in terms of power than in healthy participants. However, in some tasks, additional brain areas were activated in comparison to the healthy participants^18^.

Until now, according to our knowledge, no well-documented research results have been published regarding the assessment of the reading ability in people with minimal consciousness. The eye tracking systems have been used for the purpose of examining eye behaviour while reading in healthy subjects^19–22^. Ratiu and Azuma^23^ examined eye movements of bilingual patients after mild TBI. Some deficits in executive functioning and higher rates of language processing errors (in comparison to the healthy group) were found. Johansson *et al*.^24^ used an eye tracker as a tool for assessing the reading performance of patients after mild TBI before and after spectacle treatment. However, patients participating in that study were fully conscious and the study focused on visual dysfunction rather than on the reading comprehension and syntactic/spelling skills.

There are many unknowns and questions about the possibilities of organizing an interaction of people with severe brain injuries with assistive systems, therefore, research in this field is necessary. Its results can help develop solutions in the field of HCI systems, intended for diagnosing and supporting these people. Therefore, the main purpose of the study was to assess whether it is possible to communicate with MCS patients through the written word, employing the eye-tracking technology, and what are the aspects of this communication in the context of syntax and spelling. Bidirectional communication was taken into consideration, i.e., both the ability to comprehend the written word and the ability to arrange letters into words and words into sentences, in order to answer the guardian’s/therapist’s question, were assessed. We hypothesised that at least some patients would show residual letter and word reading abilities. We wanted to check if those patients preserved simple syntactic and spelling skills. Other hypotheses were that the patients with a higher level of motivation would perform the tasks better, and, similarly, the performance of the tasks would be better in the group of patients with intact ability to maintain attention.

## Methods

As part of planning the research, the consent of the Bioethics Committee of the Nicolaus Copernicus University in Toruń functioning at Collegium Medicum in Bydgoszcz was requested (permission to conduct the study No. KB 654/2014 dated on 2014, No. 11). The research was carried out in the Neurorehabilitation Centre of the “Epimigren” Medical Centre in Osielsko, Poland, and in the Care and Therapy Institute of the “Light” Foundation in Torun (Zakład Opiekuńczo-Leczniczy Fundacji “Światło”), Poland. All experiments were performed in accordance with relevant guidelines and regulations. Written informed consent for participation was obtained from the legal guardians of all participating subjects.

### Characteristics of subjects

Fifty-five patients who met the following inclusion criteria were recruited for the study: Non-TBI or TBI that originally led to coma; emerged from a coma to MCS. The patients who met the following exclusion criteria were not qualified for the study: severe hearing disorder – 0 subjects, neurogenic visual impairment (damage to the optic nerve, visual intersection, visual path and visual areas in the occipital lobe) – 3 subjects, damage to the extraocular muscles or cranial nerves that innervate the eyeball (nerve III, IV, VI) – 0 subjects, deep disorder of speech comprehension revealed in the “speech comprehension test” described in the next section – 2 subjects. The clinical data of the resulting 50 MCS patients (15 women) qualified for the study have been contained in Supplementary Table S1. The diagnosis of all the patients was up-to-date (not older than one month). The patients recruited for the study, who had been diagnosed more than a month before, were re-diagnosed by a neurologist confirming the same diagnosis. All the patients showed unimpaired visual pursuit when assessed at the bedside. Patients with hemispatial neglect were not participating in the study. Therefore, the patients were equally able to maintain their gaze on the objects no matter where the objects were localized on the screen. However, none of the patients was able to communicate either using oral, written (via standard means) language or gestures of any kind (e.g. yes/no head movements).

Patients with different aetiologies and time since onset were participating in the study. However, every patient was diagnosed with the same DoC, i.e., MCS. The average age of the patients was 34.2 years (minimum age was 18 years, maximum was 57 years). The standard deviation constituted 27.8% of the average value, confirming the large age diversity of the patients. The average age of women at the time of the event was less than 37 years, with the average age of men being just over 31 years. There was no statistically significant difference between men and women regarding their age (p = 0.102, Mann-Whitney U test). Twenty-seven patients (54%) had secondary education, 18 patients (36%) had higher education, and 5 patients (10%) had basic education.

In 27 patients (56%) the cause of a coma was traumatic brain injury – 24 patients (48%) acquired brain injury during a road accident and 3 patients (6%) acquired an injury from the fall. The group of remaining 23 Non-TBI patients was more diversified, with causes such as stroke (5 patients – 10%), myocardial infarction (3 patients – 6%), inhalation of water (2 patients – 4%), venous embolism (2 patients – 4%), diabetes, childbirth, heart transplant, an aneurysm, a gunshot, an antidepressant overdose, a suicide attempt, a craniotomy, haematoma, a kidney transplant, and the massage (the patient lost consciousness soon after reporting pain during the cervical vertebrae massage) (each cause represented by 1 subject – 2%).

The average time from the moment of the event to the time of the study amounted to less than 39 months. A standard deviation was 108% of the average value, which indicated a very large variation. On average, a longer time was recorded in the group of women, with the average value of approximately 51 months, and with the average in men of approximately 27 months. The shortest time reported for a single subject (male) was 3 months and the longest was 12 years (female). There were no specific reasons to look for sex differences apart from providing sociodemographic data.

### Research tools

The research was conducted using two computer programs controlled by an eye tracker. The programs – “Speech Comprehension Test” and “the Written Word Comprehension Test” – are described in the following sections. Spaces between the controllable elements on the screen and the elements themselves were big enough to ensure reliable controllability by patients, i.e., the patients could indicate the objects (i.e., words, letters or textboxes) by maintaining their gaze on them for at least 2 seconds. Moreover, developing the interface that way eliminated the need for utilizing an eye-tracker of an extremely high sampling frequency, accuracy, and precision, which in patients with minimal consciousness would not have been advantageous anyhow. Thus, any typical, commercial eye-tracker could be used for replicating the study. A dot representing a gaze fixation point was visible on the screen. Thus, a visual feedback was provided to the patient and the therapist could assess saccade movements and gaze fixation within the task 3, described in the further section.

#### Speech Comprehension Test

Speech Comprehension Test was a proprietary tool prepared for the needs of the study presented in this work. The test consisted of tasks checking answers to a simple question (Table 1) and was prepared by a speech therapist in accordance with the standard protocol of the speech comprehension test used by speech therapists. In the task 1 there was a pictogram of a thumb up presented on the left side of the screen and a pictogram of a thumb down presented on the right side, for yes and no answers. The task of the patient was to indicate by gaze the pictogram associated with the correct answer. In the next two tasks three pictures were presented to the patients and the task was to indicate the one associated with the name spoken by the therapist. Thus, comprehension of particular words (nouns and verbs), spoken by the therapist, was examined. Only the correct performance of at least 2 of the 3 tasks qualified the patient for the study. Due to the usually severe general condition of people with brain injuries, the speech comprehension tasks maintained a simple character (Table 1).

**Table 1:** Tasks in the Speech Comprehension Test.

No.	Task	Content of the task	Correct answers
1.	Answer the question	Do dogs bark?	YES (a pictogram with a thumb up is to be selected by gaze)
		Are bananas straight?	NO (a pictogram with a thumb down is to be selected by gaze)
2.	Select the image of which the name you hear	Three pictures – nouns: • chair • cow • ball-pen	chair – the correct answer (a choice made by maintaining gaze for 2 seconds on the object)
3.	Select the image of which the name you hear	Three pictures – verbs: • drink • sleep • sit	sleep – the correct answer (a choice made by maintaining gaze for 2 seconds on the object)

#### The Written Word Comprehension Test

**Figure 1:**
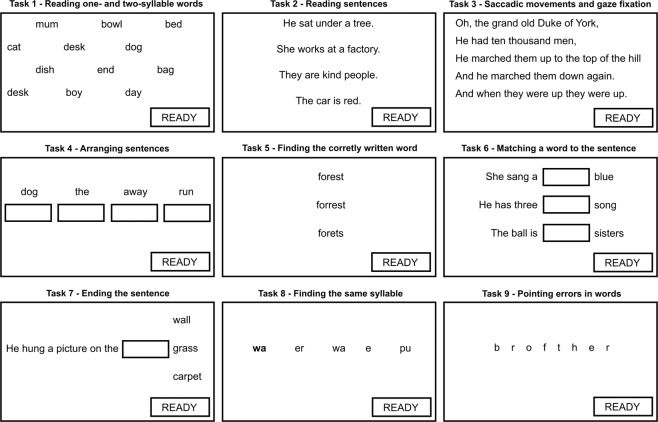
Examples of the tasks within the Written Word Comprehension Test. The patient selects the object (a word or a letter) by maintaining gaze on it for at least 2 seconds. Placing a word in the box is performed in the same manner.

Before conducting the study a group of healthy 15 people (therapists and psychologists) had checked if the precision and accuracy of the employed eye-tracking device, as defined by Tobii^25^, were sufficient to provide smooth operating, i.e., if it was possible to select every selectable object within the graphical user interface by keeping sight still on it for at least 2 seconds, considering its size and position on the screen (Fig. 1). No technical issues were reported by that group of testers and thus the system was accepted for usage with the patients.

Efforts were made to create favourable conditions for research, i.e., low noise, optimally darkened room, a small number of distractions. To standardize the tasks all subjects heard the same command in a particular task. When the subject considered the task to be finished, he or she directed his/her sight to the READY sign. The duration of the whole study was about 100 minutes. Due to the general condition of the patients, it was divided into three sessions, each lasting approximately 30 minutes. Before the first session an interview with the patient’s guardian, concerning demographic data, was held and a clinical evaluation was carried out.

The tasks of the test, used for assessing the reading ability, are described beneath. The authors made an effort to eliminate a subjective assessment during the study. Therefore, for every task, a protocol was prepared, in cooperation between a clinical therapist, a speech therapist, and psychologists. According to the protocol (see: Supplementary Tables S2 and S3), the patient was scored with a particular number of points (1–5), given for performing the task in the exactly specified manner, e.g., in the task 4 the patient was scored with 5 points for arranging two (out of two) sentences correctly, 4 points for arranging one sentence correctly and arranging two words in proper order in the next sentence, 3 points for arranging one sentence only, 2 points for beginning and ending the sentence correctly but with the wrong order of words inside, and 1 point for arranging the words in the order not meeting any of the above-mentioned conditions. A particular number of points was associated with the particular level of impairment, i.e., 5 points – ability preserved (P), 4 points – light impairment (L), 3 points – moderate impairment (M), 2 points – severe impairment (V), and 1 point – ability ceased (C). As there were nine tasks, the patient could score in total the minimum of 9 points and the maximum of 45 points. Thus, the minimum and the maximum values denoted edges of the whole range of points falling to the particular level of impairment, with values 18, 27, and 36 being the centres of ranges associated with the V, M, and L levels, respectively. Those values were obtained by multiplying the number of tasks by the number of points assigned to the particular level in a single task (e.g., 9 × 3 for the moderate impairment). Scoring was performed by the clinical therapist accompanied by another specialist (a speech therapist) to ensure there was no human error during the process. After each task, particular number of points (1–5) was written down on a printed questionnaire by the therapist. A role of the accompanying speech therapist was to observe the process and check on the fly whether the scores were given in accordance with the protocol. In case of a mistake, the speech therapist was to react immediately and inform the therapist about it. However, during the process there were no mistakes reported. The structured observation related to the assessment of motivation and to the ability to maintain attention was made during each session, also according to the strict protocol. The procedures of the assessment have been presented in Supplementary Tables S4 and S5.

Task 1 – Reading one- and two-syllable words (reading comprehension skills)

Various words are presented on the monitor (Fig. 1). The task of the subject is to select a word spoken by the therapist by maintaining gaze on it for at least 2 seconds. Selection of the word is confirmed by indicating the READY button. The process is repeated three times with the same word set.

Task 2 – Reading sentences (reading comprehension skills)

Four sentences are displayed on the screen (Fig. 1). The task of the subject is to maintain his/her gaze for at least 2 seconds on the sentence read out loud by the therapist. The process is repeated three times.

Task 3 – Saccadic movements and gaze fixation

Five lines of text are presented on the screen (Fig. 1). The task of the subject is to read silently the text. The way how a gaze is being moved between the words and the ability to fixate the gaze for at least 2 seconds are assessed. The process is performed once.

Task 4 – Arranging sentences – building complex statements (syntactic skills)

The task of the subject is to read words displayed in a line in random order, and then to arrange the words to create a proper sentence obeying grammar rules (Fig. 1). Arranging the words is performed by placing them into the textboxes displayed beneath. Choosing a particular word is performed by maintaining gaze on it for at least 2 seconds. Placing a chosen word into the textbox is performed in the same manner. Two sets of words are presented for each subject, i.e., two sentences are to be formed.

Task 5 – Finding the correctly written word (spelling, phonemic hearing)

Three words are presented on the screen, but only one word is written correctly (Fig. 1). The task of the subject is to select this word by maintaining his/her gaze on it for at least 2 seconds. The process is repeated three times (with different words).

Task 6 – Matching a word to the sentence (reading comprehension skills)

Three sentences without the last word (noun) are presented on the screen (Fig. 1). Instead of the last word, a textbox for placing the word is displayed. The missing three words are displayed next to the textboxes, in random order. The task of the subject is to read the sentences and to match the words to them. Both selecting the word and placing it in the textbox is performed by maintaining gaze on the word/textbox for at least 2 seconds. The process is performed once.

Task 7 – Ending the sentence (reading comprehension skills)

One sentence without the last word is presented on the screen (Fig. 1). The textbox for the word to be placed is displayed at the end. Three words are displayed next to the textbox. Only one word fits the end of the sentence. The task of the subject is to select this word by maintaining gaze on it for at least 2 seconds and to place the word in the textbox (in the same manner). The process is repeated 3 times (with different sentences).

Task 8 – Finding the same syllable (visual perception, spelling)

A syllable is placed on the left side of the screen (Fig. 1). Next to the syllable a group of four syllables is displayed. The task of the patient is to recognize the syllable placed on the left side of the screen in the group of four syllables and to select it by maintaining gaze on it for at least 2 seconds. The process is repeated 3 times (with different sets of syllables).

Task 9 – Pointing errors in words (visual memory of the word, spelling)

An incorrectly written word is presented on the screen (one additional letter is placed) (Fig. 1). The task of the subject is to select this letter by maintaining gaze on it for at least 2 seconds. The process is repeated three times (with different words).

### Statistical analysis

Kruskal-Wallis test and Tukey’s honest significance *post hoc* test were used for comparing the differences between the categorical groups of levels of impairment of ability to read. The null hypothesis in the Kruskal-Wallis test was that the data within the four groups of the level of impairment (V – severe, M – medium, L – light, P – preserved), came from the same distribution. As each level was represented by values from a different range it was known *a priori* that the distributions varied. Therefore, the purpose of the statistical analysis was rather to check to what degree the collected data supported the alternative hypothesis in the *post hoc* test (i.e., if the *p*-value was below 0.001, 0.01, or 0.05). The null hypothesis in the Tukey’s honest significance *post hoc* test was that means being compared between the chosen groups were from the same population. The same procedure was employed in comparing the differences between 9 categorical groups of measures of motivation (3 sessions per each of 3 measures of motivation). The null hypothesis in the Kruskal-Wallis test, used in the assessment of motivation, was that the patients were equally likely to perform identically in each of the 3 measures of motivation and in each of the three sessions. An increasing trend was observed in the results of the assessment of motivation between the sessions. Therefore, additionally, the results from the three sessions were grouped within each measure of motivation, and the Kruskal-Wallis test with the Tukey’s honest significance *post hoc* test were applied again to reveal the statistical significance of the differences between the three groups of the measures of motivation, irrespective of the session number.

Two-sided Fisher’s exact test was used for comparing the differences in a distribution of the number of patients falling within each categorical group of the level of motivation, measured in three sessions. The applied procedure consisted in comparing the number of patients, separately in each session and between each two of the three levels (A – anti-motivation, M – moderate motivation, H – high motivation). Therefore, for each compared pair a 2 × 2 contingency matrix was created with one row adopting values conforming to the uniform distribution of patients between the motivation levels and another row adopting the actual numbers of patients. 50 patients took part in sessions I and II, and 48 patients took part in session III (two of the 50 patients completed all the tasks in sessions I and II, and thus they did not participate in session III). Thus, the uniform distribution of patients between the categorical groups of the level of motivation was represented by values 17, 17, and 16, for sessions I, II, and III, respectively. The same approach was applied to the comparison of the differences between the number of patients across categorical groups of the level of ability to maintain attention. The null hypothesis in both cases was that the patients were equally likely to fall within each group corresponding to the particular level. The two-sided Fisher’s exact test was also used for the purpose of analysing the distribution of patients amongst the grouped levels of ability to read in each of the 9 tasks, as described beneath.

The authors had previously considered an approach in which no levels were distinguished and trends of the results were to be analysed exclusively based on the given numerical scores. However, that approach was criticized by psychologists with whom the study design was consulted and who strongly encouraged using levels easy to understand by the reader. Therefore, after completing all the sessions with patients the obtained scores were grouped by levels, specified in collaboration between a clinical therapist, a speech therapist, and psychologists, based on the observations made during the sessions and based on the protocol according to which the tasks were scored, as described earlier in section 2.2.2. As there was a clear tendency in the scores given in the tasks to concentrate either on the side of the P (preserved) level or on the side of the C (ceased) level, with fewer instances among the middle levels (V, M, L) we applied an approach in which the results from levels C, V, M constituted one group and the results from levels L, P constituted another group. The numbers of patients were compared in 2 × 2 contingency matrices with the values corresponding to the uniform distribution amongst the five levels, i.e., the value of 30 for the C, V, M group and the value of 20 for the L, P group. The null hypothesis was that the patients were equally likely to fall within those two groups (considering the 30/20 proportion). That approach enabled to analyse the results without comparisons between the neighbouring levels. As the sharp thresholds were used (e.g., score of 40 fell into the P category and score of 39 fell into the L category) applying the two-sided Fisher’s exact test in such comparisons between the neighbouring levels could have been considered misuse. The applied grouping enabled to “smoothen” the sharp transitions, still, revealing significant differences between the two groups. The same approach was employed in checking the statistical significance after grouping the patients together within all the tasks.

Mann-Whitney U test was used for checking the statistical significance of the differences between the age of male and female subjects, the results of the assessment of motivation in groups of male and female subjects, the results of the assessment of motivation in groups of a cause of a coma, the results of the assessment of the ability to maintain attention in groups of male and female subjects, and the results of the assessment of the ability to maintain attention in groups of a cause of a coma. The null hypothesis in each case was that the randomly selected sample from one of the compared groups was equally likely to be smaller or greater than the randomly selected sample from another group.

The Spearman’s rank correlation coefficient was calculated in order to check the correlations between the motivation and the level of the reading ability, between the ability to maintain attention and the level of the reading ability, between the motivation and the ability to maintain attention, and between the sociodemographic data and motivation and the ability to maintain attention. The Spearman correlation was also used for checking internal correlations between the nine tasks.

## Results

**Figure 2:**
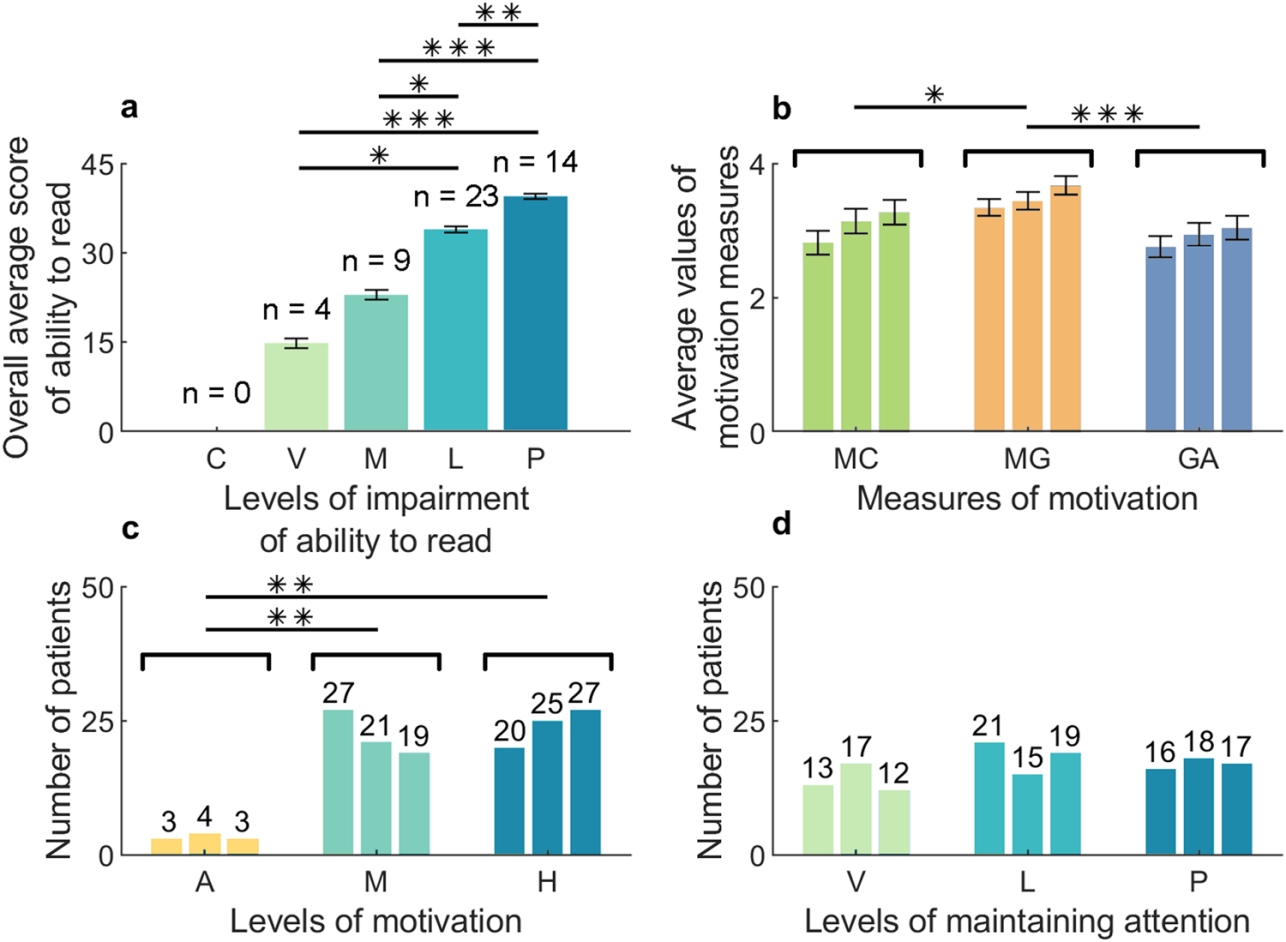
The results of the assessment of the reading ability impairment in 50 post-comatose patients, analysed taking into account their motivation and ability to maintain attention. (**a**) Overall average scores of the patients in each level of the reading ability impairment. In none of the patients the ability was completely ceased (*p < 0.05, **p < 0.01, ***p < 0.001, Kruskal-Wallis test and Tukey’s honest significant difference post hoc test); C – ability completely ceased, V – severe impairment, M – moderate impairment, L – light impairment, P – ability preserved. (**b**) The average values of the measures of motivation in three sessions. An increase of motivation, in all three measures, was observed in consecutive sessions. The differences between the results of MC (muscle contraction) and the results of MG (maintaining gaze) were statistically significant (*p < 0.05), as well as the differences between the results of MG and the results of GA (general activity) (***p < 0.001) (Kruskal-Wallis test and Tukey’s honest significant difference post hoc test). (**c**) Histogram with the number of patients classified to one of the three levels of motivation, in each session. Only 3 patients in sessions I and III and 4 patients in session II were anti-motivated to perform the tasks (two-sided Fisher’s exact test, **p < 0.01). Notice a gradual change of motivation from moderate to high with consecutive sessions. A – anti-motivation, M – moderate motivation, H – high motivation. (**d**) Histogram with the number of patients classified to one of the three levels of deficit of attention. The differences were not statistically significant, both in terms of the level and the session number (two-sided Fisher’s exact test). V – severe deficit; L – light deficit, P – attention properly maintained.

All 50 patients participated in sessions I and II. Forty-eight patients participated in session III as two patients completed all the tasks within the sessions I and II, and thus they did not participate in the last session. The average score of the reading ability in that group of patients was 33.94 points. As presented in Supplementary Table S3, it is a result of a slight impairment of the reading ability, on the verge of the moderate impairment. The standard deviation was 22.7% of the average value, which indicated a large differentiation of the scores. The minimum score was 16 points and the maximum was 45 points, meaning full preservation of the reading ability. According to the procedure presented in Supplementary Table S3, the results were grouped by five levels of the reading ability impairment (Fig. 2a). Most patients (23 subjects – 46%) had the reading ability slightly impaired (L). Only 4 patients (8%) had the reading ability vastly impaired (V) and in none of the patients the ability was completely ceased (C).

Regarding motivation of the patients to perform the tasks, the highest average value of the results from the three sessions was obtained for a measure representing maintaining a gaze (MG), and the smallest one was obtained for a measure representing general activity (GA) (Fig. 2b, Supplementary Table S4). That relation was consistent also for partitive results of the three sessions. The differences between the average values for the muscle contraction measure (MC) and for the MG, as well as the differences between the average values for the MG and for the GA, were statistically significant (Kruskal-Wallis test and Tukey’s honest significant difference post hoc test; n = 50 in sessions I and II, n = 48 in session III; p = 0.032 in MC–MG, p = 0.0001 in MG–GA) (Fig. 2b). Moreover, an increase of motivation was observed in consecutive sessions, although that trend was not statistically significant (Kruskal-Wallis test; n = 50 in sessions I and II, n = 48 in session III; p = 0.13 in MC, p = 0.24 in MG, and p = 0.48 in GA) (Fig. 2b). It was checked that the age, education, and time that elapsed since the day of an injury were not in a statistically significant correlation with motivation (Spearman correlation, p = 0.897, p = 0.851, p = 0.371, respectively). It was also checked that the differences between the results of the assessment of motivation in groups of male and female subjects, as well as the differences between the results of the assessment of motivation in groups of a cause of a coma, were not statistically significant (Mann-Whitney U test, p = 0.720, p = 0.238, respectively). Based on that finding one could presume that a trend of the obtained results of the assessment of motivation (Fig. 2b) would stay intact also for a higher population of patients. The exact scores of every patient in the three measures of motivation, in each session, have been presented in Supplementary Table S7.

A statistically significant moderate correlation between the motivation and the reading ability has been found (Spearman correlation, R = 0.3, p = 0.03). Higher scores of the reading ability were recorded in the patients with higher motivation. Three patients in sessions I and III and four patients in session II were anti-motivated to perform the tasks (Fig. 2c). They turned their heads to avoid looking at the monitor screen or kept closing their eyes (although their eyes were open before and after the sessions).

The differences in the distribution of the number of patients classified to one of the three levels of maintaining attention (see: Supplementary Table S5), between the sessions, were not statistically significant (two-sided Fisher’s exact test, p = 0.72). By averaging the results from the sessions it was stated that most patients preserved or had slightly impaired ability to maintain their attention on the tasks (17 and 21 patients, respectively). A severe deficit of attention was recorded in 12 subjects. The results of the assessment of the ability to maintain attention, for every patient, in each session, have been presented in Supplementary Table S8. They were not in a statistically significant correlation with the reading ability (Spearman correlation, p = 0.08). However, the low *p*-value may indicate an importance of conducting therapeutic sessions with patients with minimal consciousness, aimed at enhancing their attention and concentration. A highly significant positive correlation between maintaining attention and motivation was found (Spearman correlation, R = 0.53, p = 0.0008). It was checked that age, education, and time that elapsed since the day of an injury were not in a statistically significant correlation with the ability to maintain attention (Spearman correlation, p = 0.366, p = 0.096, p = 0.177, p = 0.371). It was also checked that there were no statistically significant differences between sex (i.e., male versus famale), nor between aetiologies (i.e., TBI versus Non-TBI) (Mann-Whitney U test, p = 0.43, p = 0.177, respectively).

**Figure 3:**
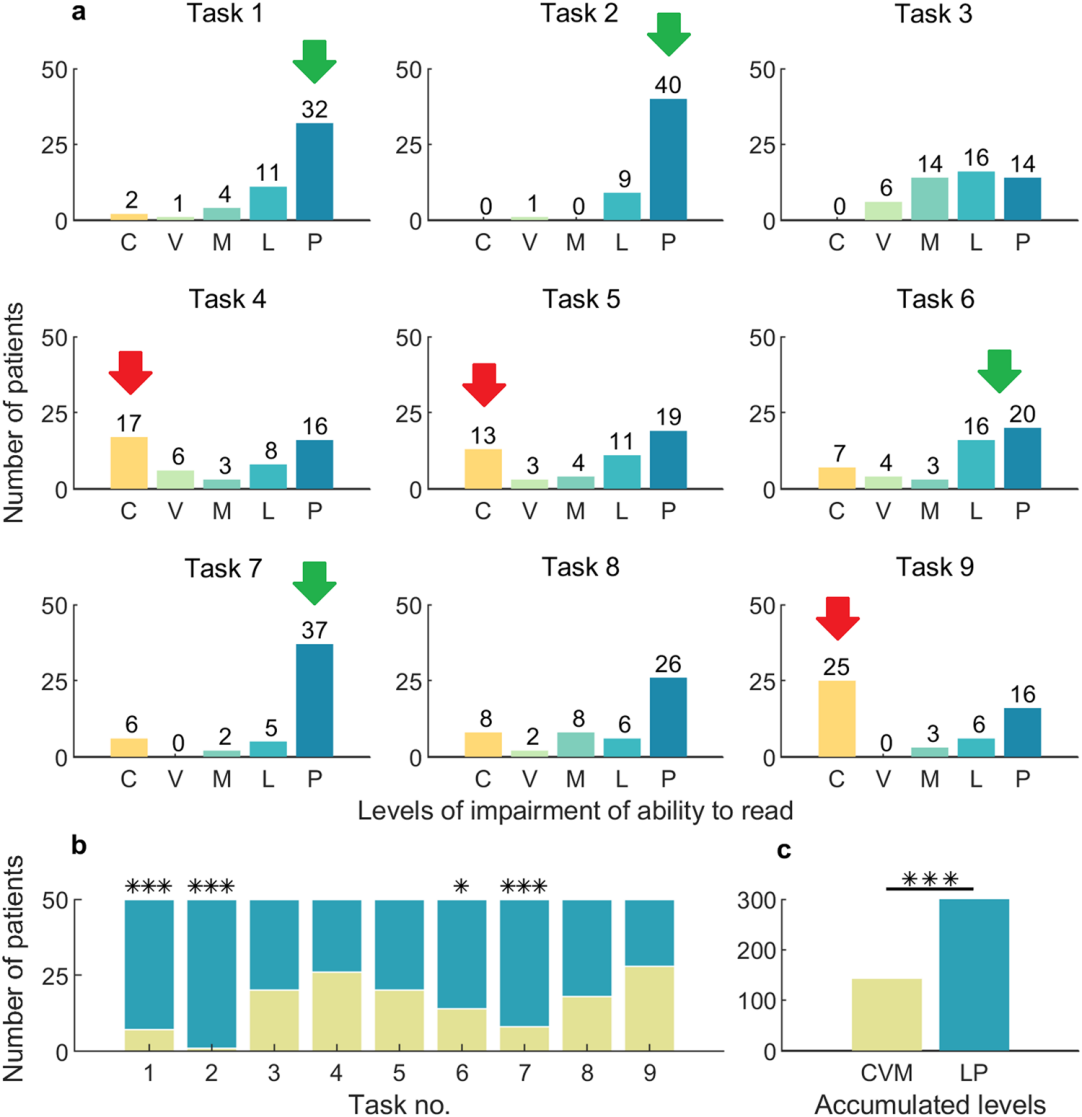
Reading comprehension skills in the group of 50 post-comatose patients, in the 9 tasks. (**a**) Histograms with the number of patients in each group of five levels of the reading ability impairment, for the 9 tasks of “the Written Word Comprehension Test”. Most patients preserved the reading comprehension skills (green arrows) but had poor ability to recognize errors in sentences and words (red arrows). (**b**) The differences between the accumulated number of patients with the C, V, M levels and the L, P levels were statistically significant in the tasks 1, 2, 6, and 7 (*p < 0.05, **p < 0.01, ***p < 0.001, two-sided Fisher’s exact test). (**c**) The difference between the ac**c**umulated number of patients with the C, V, M levels and the L, P levels, within all the tasks grouped together (***p < 0.001, two-sided Fisher’s exact test). C – ability completely ceased, V – severe impairment, M – moderate impairment, L – light impairment, P – ability preserved.

It has been shown that most patients (86%) had a completely preserved or slightly disturbed ability to read one- and two-syllable words (Fig. 3a). Relatively correct performance was also observed in the task 2 (“reading sentences/reading comprehension skills”) (80% performed correctly – Fig. 3a), and in the task 7 (“ending the sentence/reading comprehension skills”) (74% performed correctly – Fig. 3a). In all three, above mentioned tasks, used for assessing the reading comprehension skills, the differences between the accumulated number of patients with the C, V, M levels and the L, P levels were statistically significant (Fig. 3b, two-sided Fisher’s exact test, p < 0.001). In the task 4 (arranging sentences – building complex statements/syntactic skills), a total disintegration of grammar occurred in 34% of the examined subjects, but 32% of the patients had preserved the ability to build complex statements. Only 50% of the patients completed the task 9 (“pointing errors in words/visual memory of the word, spelling”) (Fig. 3a). Correlation between the task 9 and the task 3 (saccadic movements and gaze fixation) was checked, but no statistical significance was demonstrated (Table 2). On the other hand, a correlation was found between the visual memory of the word (task 9) and the ability to recognize errors in the written text (task 5), i.e., the ability increased with an increase of the score of the visual memory of the word (38% of the patients performed the task 5 correctly and 22% had the ability slightly disturbed – Fig. 3a). A significant influence of the saccadic movements and gaze fixation, assessed in the task 3, on the proper execution of the task 5, was also observed (Table 2). The highest scores of the ability to recognize errors in the written text were recorded in the group of patients with preserved or slightly disturbed saccadic movements and gaze fixation. The post-comatose patients with minimal consciousness obtained, in a clear majority, the results indicating the preserved or slightly disturbed reading ability (Fig. 3c).

**Table 2:** Internal correlations between the tasks used for assessing the reading ability; significant correlations (Spearman correlation, p < 0.05) highlighted in bold.

Task 2	**0.41**								
Task 3	−0.02	0.28							
Task 4	0.08	0.20	0.19						
Task 5	−0.09	0.14	**0.41**	**0.36**					
Task 6	0.09	0.22	**0.40**	**0.69**	0.29				
Task 7	0.18	**0.31**	**0.40**	**0.43**	**0.37**	**0.57**			
Task 8	0.02	0.17	0.06	**0.31**	0.28	**0.31**	0.22		
Task 9	*-*0.09	0.35	0.16	**0.49**	0.40	**0.34**	0.26	**0.37**	
**Task No**	**Task 1**	**Task 2**	**Task 3**	**Task 4**	**Task 5**	**Task 6**	**Task 7**	**Task 8**	**Task 9**
Correlation with reading ability	0.09	**0.35**	**0.42**	**0.75**	**0.66**	**0.66**	**0.57**	**0.48**	**0.67**

## Discussion

The patients willingly performed the tasks, which may indicate an urge of people with consciousness impairments to communicate with the environment. That confirms that even after a severe brain damage, language functions can be preserved. In the presented study, none of the patients had a completely ceased reading ability. It was confirmed partially what is known from the literature, i.e., that people with brain injuries may have syntactic disorders^26^. Most patients preserved the ability to read one- and two-syllable words and preserved comprehension of sentences but had poor ability to recognize errors, both in the words (wrong letters) and in the sentences (wrong order of words in a sentence). However, errors deliberately inserted into the words by the therapist did not affect the reading comprehension in that group of patients. This finding is consistent with a well-known phenomenon, observed in healthy adults and children, and first described by Graham Rawlinson^27^ and later studied by others^28–32^, also with a focus on an eye-fixation behaviour^33^. According to that phenomenon, provided that the rules specified by Rawlinson and extended later by the authors of the above-mentioned studies are met, comprehension of garbled text (e.g., text with letter positions changed in a word) is similar to comprehension of the text written correctly. Observation had also indicated that the patients performed the task 1, aimed at assessing the ability to read one- and two-syllable words, noticeably faster than the other tasks, still preserving a high level of correct answers. This observation supports the above-mentioned feature of reading (i.e., the phenomenon observed by Rawlinson^27^), which seemed to be preserved to a high extent in that group of post-comatose patients. An impaired ability to recognize misspellings might indicate impairments in the brain areas responsible for shape processing while reading employing letter decoding strategies.

People with acquired brain injuries often have various deficits in attention^34^. Results of other research show that even slight deficits of attention may cause an impairment of other functions^35^. In our study, three relatively uniform groups of patients were distinguished, characterized by a proper maintaining of attention, a light deficit of attention, and a severe deficit. Patients with a moderate deficit of attention were not identified. Although there was no statistically significant correlation between the ability to maintain attention and the ability to read, a highly significant correlation was observed between the ability to maintain attention and the motivation, which in turn was in a statistically significant correlation with the ability to read.

Results of numerous studies show that time since onset has an impact on chances of regaining consciousness^36–38^. If the subject does not regain consciousness within three months after cerebral hypoxia or in less than a year after the craniocerebral trauma, the chances of a brain damage reversibility are very small. Typically, the mentioned intervals are even set as the limits of intensive rehabilitation. However, in our study, no significant differences were found between the time since onset and the obtained results of the reading skills. What is even more surprising, the best results of that task were obtained in the group of patients who acquired an injury at least three years earlier, and the worst results – in the group of patients who had an injury less than a year earlier. That finding seems to be not consistent with assumptions related to the Neural Darwinism theory, according to which a disintegration of unused synaptic pathways and a deterioration of cognitive functions should have occurred^39^. This is however in agreement with a spontaneous recovery linked to the neural plasticity, what could occur even in chronic DoC patients, as previously demonstrated^40^. Therefore, the results of our study show that time since onset should not be treated as the determinant in diagnosis and in a rehabilitation process.

The obtained results provide hints for developing gaze tracking-based human-computer interfaces for people with minimal consciousness. The first hint is associated with the spelling negligence and concerns advice to replace words that are graphically similar to other words of a different meaning (e.g. *coarse* and *course*) with their synonyms (e.g. *rough* instead of *coarse*). Also, due to the syntax negligence, commands displayed within the graphical user interface should be as simple and short as possible to avoid ambiguity resulting from impaired perception.

There is a lot of controversy regarding the choice of a right communication model describing a disappearance of the ability to read, i.e., alexia. Among the best-known classifications of alexia^41^ are pure alexia (mainly a visual disorder), surface alexia (a disorder of semantics), and phonological alexia (a phonological processing deficit). People suffering from pure alexia, after adapting to their disability, are able to identify and name individual letters over time as well as recognize sequences of letters as words. Thus, they are able to read but the process takes longer than in healthy people. The difficulties arise with an increasing word length. Other language activities, e.g. writing, are unaffected. Research suggests that pure alexia is caused by deficits at a higher level in the visual processing form^42^. The term “surface alexia” (or “surface dyslexia”) is used to describe problems in reading of irregular words (not consistent with their spelling) with a preserved reading of regular words (consistent with their spelling) and nonwords. Conversely, patients with phonological alexia (phonological dyslexia) can read regular and irregular words but may have difficulties in reading nonwords. All the mentioned types of alexia are caused by damages in different brain areas. Ripamonti *et al*.^43^ have found that pure alexia is associated with lesions in the left fusiform gyrus, surface alexia is associated with the left temporal lesions, while in phonological alexia the lesions are overlapped in the left insula and the left inferior frontal gyrus. Most patients in our study were able to read words but neglected spelling. Therefore, as they had not performed any exercises related to reading before the study and thus they had had no opportunity to create new reading strategies to overcome deficits, it could be stated that the reading ability was not affected by pure alexia. Moreover, the ability to form a word or a sentence was severely impaired in most patients whereas in pure alexia the ability to write is preserved. One could hypothesise that the lack of ability to find errors in words was an indicator of phonological alexia, as the incorrectly written words formed non-words and thus were unreadable. However, the patients were not able to identify the correctly written word among the non-words as well and that would indicate impairments of another type than phonological alexia. Specifying the exact type of alexia in MCS patients is a challenge because of a lack of verbal communication and a presence of impairments of various kinds which combined may mimic a particular type of alexia.

As the commands were given verbally by the therapist, the limitation of the study is that our HCI approach in its current form could be used only with patients who comprehend speech or at least have residual comprehension abilities. Although it would be a rare case, one can imagine a scenario in which a patient preserved the reading comprehension skills but does not comprehend speech. In such a case, commands should be provided in the written form within the graphical interface. However, fitting the written commands within the screen would demand to decrease the font size of the task contents. In consequence, using the interface by gaze might get difficult or impossible. Regarding the control of the application via gaze, one can also consider MCS patients with hemispatial neglect. For such patients the contents of the tasks should be displayed in the particular side of the field of vision. This might be accomplished either by displaying the contents on a particular side of the screen or placing the screen in a particular side of the field of vision, provided that the patient’s head is kept straight ahead. In the first case, a large format display would be used. In the second case, a special mounting for an eye-tracker should be employed enabling its positioning outside the monitor. Another limitation concerns using the system with the MCS *minus* patients, according to subcategorization of the MCS introduced by Bruno *et al*.^44,45^. Such patients might not be able to follow the commands given by a therapist due to the ceased cerebral metabolism in the left-sided cortical areas, including Broca’s area and Wernicke’s area^46^. The MCS *minus* patients present reduced residual language abilities as compared to the MCS *plus* patients^46^. Another issue is related to within-day variability of a state of the MCS patients^47,48^, i.e., performance in the tasks can vary depending on the time of day. Also, when scoring the patient responses it is a common practice that the scorers are blinded to the patient identities and conditions as otherwise they are likely to be biased. In our study, scoring was performed by the non-blinded clinical therapist accompanied by the speech therapist, as described in the Methods section. The protocol allowed for giving a particular number of points only if a particular operation within the task was completed. The role of the speech therapist was to observe the whole process and react in case of a mistake or a tendency of the clinical therapist towards bias. In none of the cases the speech therapist needed to intervene. Still, some might argue that the observer might have been sluggish or timid with the reactions. Also, while replicating the study one might consider changing the order of presentation of the tasks to avoid the decrease of attention. In that study the trials within each task were performed sequentially, i.e., they were not separated by the trials of the other tasks.

The patients were recruited from many medical centres around the country. They had been diagnosed in those medical centres and based on the diagnosis, i.e., MCS (and no spatial neglect), they were qualified for the study. Information about how the diagnosis was performed, i.e., whether the CRS-R scale or other scales were used, was not provided to the authors. Without the use of standardized behavioural scales such as the CRS-R, the rate of patients’ misdiagnosis reaches approximately 41%^7^. In study by Schnakers *et al*.^7^ it was shown that using the CRS-R revealed 41% of patients, originally diagnosed with a vegetative state based on the clinical consensus, to be in the MCS. 10% of patients with a consensus diagnosis of the MCS had emerged from the MCS, according to the CRS-R^7^. In our study, no patient showed a completely ceased reading ability, which indicate there were no UWS patients in that group. Still, none of the patients was able to communicate verbally or through yes/no head movements.

In conclusion, the developed HCI system enabled to assess the reading comprehension skills in the post-comatose MCS patients, revealing a preserved or slightly disturbed ability to read words and sentences in most patients. Simultaneously, those patients neglected syntax what manifested in lack of ability to build sentences obeying grammar rules, and neglected spelling of words. Therefore, while it is possible to convey a message to the MCS patients through the written word one should not expect them to reply using the same means. The results of the study show the legitimacy and importance of transforming medical practice in such a way that emerging technologies could be employed as tools supplementary to the currently used clinical approaches. Combining well-grounded methods with eye-tracking devices could give better insight into a patient’s condition and in consequence contribute to the development of new therapeutic methods.

## Supplementary information

Supplementary Information

## Data Availability

The datasets generated and analysed during the current study are available from the corresponding author on reasonable request.
