# Supplementary material for: Post-comatose patients with minimal consciousness tend to preserve reading comprehension skills but neglect syntax and spelling
Source: Sci Rep. 2019 Dec 27;9:19929. doi: 10.1038/s41598-019-56443-6 (PMC6934549; doi:10.1038/s41598-019-56443-6)
Supplement: Supplementary file 1 — Supplementary Information [file 41598_2019_56443_MOESM1_ESM.docx]

Post-comatose patients with minimal consciousness tend to preserve reading comprehension skills but neglect syntax and spelling

Agnieszka Kwiatkowska, Michał Lech, Piotr Odya, Andrzej Czyżewski

**Supplementary information**

**Supplementary Table S1.** Patient clinical profiles

| **No.** | **Sex** | **Age** | **Aetiology** | **Time since onset (in months)** | **Initial diagnosis after “awakening” from coma** | **Initial**  **GCS** | **Diagnosis on the day of the study** |
| --- | --- | --- | --- | --- | --- | --- | --- |
| 1 | F | 35 – 40 | Non-TBI (stroke) | 3 | UWS | 6 | MCS |
| 2 | F | 30 – 35 | Non-TBI (cerv. vert. massage) | 11 | UWS | 6 | MCS |
| 3 | F | 20 – 25 | TBI (road accident) | 16 | shallow coma | 7 | MCS |
| 4 | M | 55 – 60 | Non-TBI (stroke) | 29 | UWS | 8 | MCS |
| 5 | M | 30 – 35 | Non-TBI (craniotomy) | 24 | tetraplegia with DoC | 8 | MCS |
| 6 | M | 30 – 35 | TBI (road accident) | 14 | UWS | 8 | MCS |
| 7 | M | 20 – 25 | TBI (road accident) | 4 | UWS | 5 | MCS |
| 8 | M | 25 – 30 | TBI (road accident) | 22 | shallow coma | 7 | MCS |
| 9 | M | 30 – 35 | TBI (road accident) | 17 | shallow coma | 8 | MCS |
| 10 | F | 50 – 55 | TBI (road accident) | 50 | UWS | 6 | MCS |
| 11 | M | 30 – 35 | Non-TBI (haematoma) | 78 | UWS | 5 | MCS |
| 12 | F | 40 – 45 | TBI (road accident) | 58 | UWS | 7 | MCS |
| 13 | F | 25 – 30 | TBI (road accident) | 24 | UWS | 6 | MCS |
| 14 | F | 30 – 35 | TBI (road accident) | 114 | UWS | 6 | MCS |
| 15 | M | 40 – 45 | Non-TBI (stroke) | 9 | UWS | 8 | MCS |
| 16 | M | 30 – 35 | Non-TBI (diabetes) | 16 | UWS | 6 | MCS |
| 17 | F | 30 – 35 | Non-TBI (kidney transplant) | 48 | UWS | 7 | MCS |
| 18 | M | 45 – 50 | Non-TBI (stroke) | 23 | UWS | 6 | MCS |
| 19 | M | 25 – 30 | TBI (road accident) | 3 | tetraplegia with DoC | 9 | MCS |
| 20 | M | 40 – 45 | TBI (fall) | 4 | UWS | 6 | MCS |
| 21 | F | 55 – 60 | Non-TBI (aneurysm) | 3 | tetraplegia with DoC | 7 | MCS |
| 22 | M | 30 – 35 | TBI (road accident) | 24 | UWS | 6 | MCS |
| 23 | M | 25 – 30 | TBI (fall) | 3 | UWS | 7 | MCS |
| 24 | M | 25 – 30 | TBI (fall) | 8 | UWS | 7 | MCS |
| 25 | M | 25 – 30 | TBI (road accident) | 9 | UWS | 9 | MCS |
| 26 | M | 40 – 45 | Non-TBI (venous embolism) | 3 | UWS | 6 | MCS |
| 27 | M | 20 – 25 | Non-TBI (gunshot) | 48 | tetraplegia with DoC | 6 | MCS |
| 28 | F | 25 – 30 | TBI (road accident) | 101 | tetraplegia with DoC | 7 | MCS |
| 29 | M | 30 – 35 | TBI (road accident) | 14 | UWS | 6 | MCS |
| 30 | M | 25 – 30 | Non-TBI (myocard. infarction) | 5 | UWS | 5 | MCS |
| 31 | M | 30 – 35 | Non-TBI (antidepressants) | 7 | UWS | 8 | MCS |
| 32 | F | 35 – 40 | Non-TBI (venous embolism) | 71 | UWS | 5 | MCS |
| 33 | M | 30 – 35 | TBI (road accident) | 76 | UWS | 5 | MCS |
| 34 | M | 25 – 30 | TBI (road accident) | 103 | UWS | 7 | MCS |
| 35 | M | 20 – 25 | TBI (road accident) | 5 | UWS | 6 | MCS |
| 36 | F | 35 – 40 | Non-TBI (childbirth) | 32 | UWS | 6 | MCS |
| 37 | M | 30 – 35 | Non-TBI (inhalation of water) | 20 | tetraplegia with DoC | 7 | MCS |
| 38 | F | 45 – 50 | Non-TBI (stroke) | 84 | UWS | 6 | MCS |
| 39 | M | 25 – 30 | Non-TBI (suicide attempt) | 13 | UWS | 7 | MCS |
| 40 | F | 30 – 35 | TBI (road accident) | 5 | UWS | 7 | MCS |
| 41 | M | 35 – 40 | Non-TBI (myocard. infarction) | 5 | UWS | 7 | MCS |
| 42 | M | 35 – 40 | Non-TBI (inhalation of water) | 88 | UWS | 6 | MCS |
| 43 | M | 50 – 55 | Non-TBI (myocard. infarction) | 4 | UWS | 8 | MCS |
| 44 | M | 30 – 35 | TBI (road accident) | 8 | UWS | 9 | MCS |
| 45 | M | 15 – 20 | TBI (road accident) | 3 | shallow coma | 9 | MCS |
| 46 | M | 40 – 45 | Non-TBI (heart transplant) | 22 | tetraplegia with DoC | 6 | MCS |
| 47 | F | 35 – 40 | TBI (road accident) | 144 | UWS | 6 | MCS |
| 48 | M | 50 – 55 | TBI (road accident) | 120 | UWS | 6 | MCS |
| 49 | M | 30 – 35 | TBI (road accident) | 48 | UWS | 7 | MCS |
| 50 | M | 30 – 35 | TBI (road accident) | 49 | UWS | 7 | MCS |

**Supplementary Table S2.** The tasks are being assessed using a 5-point scale, i.e., 1 (inability to perform the task) – 5 (the task completed and performed correctly).

| **Task 1. Ability to read one- and two-syllable words** | | |
| --- | --- | --- |
| **Performance of the task** | **Assessment** | **Score** |
| The patient indicated three words correctly | Preserved ability to read one- and two-syllable words (P) | 5 |
| The patient indicated two words correctly | Slightly impaired ability to read one- and two-syllable words (L) | 4 |
| The patient indicated one word correctly | Moderately impaired ability to read one- and two-syllable words (M) | 3 |
| The patient did not indicate any of the spoken words correctly but indicated the words similar in terms of pronunciation | Severely impaired ability to read one- and two-syllable words (V) | 2 |
| The patient did not indicate any of the words | Entirely ceased ability to read one- and two-syllable words (C) | 1 |
| **Task 2. Associating verbal and auditory patterns, reading comprehension skills** | | |
| **Performance of the task** | **Assessment** | **Score** |
| The patient indicated three sentences correctly | Preserved ability to read sentences (P) | 5 |
| The patient indicated two sentences correctly | Slightly impaired ability to read sentences (L) | 4 |
| The patient indicated one sentence correctly | Moderately impaired ability to read sentences (M) | 3 |
| The patient did not indicate any of the sentences correctly but made multiple attempts | Severely impaired ability to read sentences (V) | 2 |
| The patient did not indicate any of the sentences correctly | Entirely ceased ability to read sentences (C) | 1 |
| **Task 3. Saccade movements and gaze fixation** | | |
| **Performance of the task** | **Assessment** | **Score** |
| Stable eye movements through the whole text, 2-second gaze fixation was not a problem | Proper saccade movements and proper gaze fixation (P) | 5 |
| A dot representing fixation point was moving smoothly on the screen with occasional jumps between the words and/or 2-second gaze fixation was problematic | Saccade movements and / or gaze fixation slightly impaired (L) | 4 |
| Lines of text were skipped, there were jumps between the words and / or 2-second gaze fixation was problematic | Saccade movements and / or gaze fixation moderately impaired (M) | 3 |
| Lines of text were skipped, there were jumps between the words, the beginning or end of the line was omitted and/or 2-second gaze fixation was problematic | Saccade movements and / or gaze fixation severely impaired (V) | 2 |
| The patient was not able to follow the text and fixate for at least 2 seconds | Saccade movements and / or gaze fixation completely ceased (C) | 1 |
| **Task 4. Arranging sentences – building complex statements (syntactic skills)** | | |
| **Performance of the task** | **Assessment** | **Score** |
| The patient arranged two sentences correctly | Preserved syntactic skills (P) | 5 |
| The patient arranged one sentence correctly and arranged two words in proper order in the next sentence | Slightly impaired syntactic skills (L) | 4 |
| The patient arranged one sentence correctly | Moderately impaired syntactic skills (M) | 3 |
| The patient correctly began and ended the sentence | Severely impaired syntactic skills (V) | 2 |
| The patient arranged words in the wrong order | Entirely ceased syntactic skills (C) | 1 |
| **Task 5. Ability to recognize errors in the written text (spelling, phonemic hearing)** | | |
| **Performance of the task** | **Assessment** | **Score** |
| The patient indicated three correctly spelt words | Preserved ability to recognize errors in a word (P) | 5 |
| The patient indicated two correctly spelt words | Slightly impaired ability to recognize errors in a word (L) | 4 |
| The patient indicated one correctly spelt word | Moderately impaired ability to recognize errors in a word (M) | 3 |
| The patient indicated one correctly spelt word but made multiple attempts | Severely impaired ability to recognize errors in a word (V) | 2 |
| The patient did not indicate any word properly | Entirely ceased ability to recognize errors in a word (C) | 1 |
| **Task 6. Arranging sentences (reading comprehension skills)** | | |
| **Performance of the task** | **Assessment** | **Score** |
| The patient did match correctly three words to the sentences in the first attempt | Preserved ability to comprehend sentences (P) | 5 |
| The patient did match correctly three words to the sentences after more than one attempt | Slightly impaired ability to comprehend sentences (L) | 4 |
| The patient did match one word correctly to the sentence in the first attempt | Moderately impaired ability to comprehend sentences (M) | 3 |
| The patient did match one word correctly to the sentence after more than one attempt | Severely impaired ability to comprehend sentences (V) | 2 |
| The patient did not match correctly any of the words to the sentences | Entirely ceased ability to comprehend sentences (C) | 1 |
| **Task 7. Ending the sentence (reading comprehension skills)** | | |
| **Performance of the task** | **Assessment** | **Score** |
| The patient did match the words to the sentences correctly in all three sets | Preserved ability to comprehend sentences (P) | 5 |
| The patient did match the words to the sentences correctly in two sets | Slightly impaired ability to comprehend sentences (L) | 4 |
| The patient did match one word to the sentence correctly | Moderately impaired ability to comprehend sentences (M) | 3 |
| The patient did match the word to the sentence correctly but needed multiple attempts | Severely impaired ability to comprehend sentences (V) | 2 |
| The patient did not match the words to the sentences correctly | Entirely ceased ability to comprehend sentences (C) | 1 |
| **Task 8. Visual perception and spelling** | | |
| **Performance of the task** | **Assessment** | **Score** |
| The patient indicated three syllables correctly | Preserved visual perception of the syllables (P) | 5 |
| The patient indicated two syllables correctly | Slightly impaired visual perception of the syllables (L) | 4 |
| The patient indicated one syllable correctly and indicated two syllables which contained a proper letter | Moderately impaired visual perception of the syllables (M) | 3 |
| The patient indicated one syllable correctly | Severely impaired visual perception of the syllables (V) | 2 |
| The patient did not indicate any of the syllables correctly | Entirely ceased visual perception of the syllables (C) | 1 |
| **Task 9. A graphic image of the word, visual memory of the word, spelling.** | | |
| **Performance of the task** | **Assessment** | **Score** |
| The patient found the additional letter in all three words | Preserved ability to spell (P) | 5 |
| The patient found the additional letter in two words | Slightly impaired ability to spell (L) | 4 |
| The patient found the additional letter in one word | Moderately impaired ability to spell (M) | 3 |
| The patient did not find any of the additional letters or indicated the wrong letters | Ability to spell completely ceased (C) | 1 |

**Supplementary Table S3.** Overall assessment of the reading ability based on the scores from each task, as presented in Supplementary Table S2. For the nine tasks the patient can score the maximum of 45 points.

| **Range of points** | **Score** |
| --- | --- |
| 40-45 | Preserved (P) |
| 32-39 | Light impairment (L) |
| 22-31 | Moderate impairment (M) |
| 14-21 | Severe impairment (V) |
| below 13 | Ability completely ceased (C) |

**Supplementary Table S4.** Assessment of motivation. In each session, patients could score between 3 and 15 points, in total. Anti-motivation (A) – 3-4 points; moderate motivation (M) – 5-9 points; high motivation (H) – 10-15 points.

| **Muscle contraction (MC)** | **Score** |
| --- | --- |
| The patient contracts his/her hand and/or leg muscles + neck muscles + opens his/her eyes wide + attempts to turn his/her head | 5 |
| The patient contracts his/her hand and/or leg muscles + neck muscles + opens his/her eyes wide | 4 |
| The patient contracts his/her hand and/or leg muscles + neck muscles | 3 |
| The patient contracts his/her hand and/or leg muscles | 2 |
| No reaction | 1 |
| **Maintaining gaze on the object (MG)** |  |
| The patient maintains his/her gaze on the monitor during the whole 30-minute session | 5 |
| The patient closes his/her eyes and/or turns his/her head avoiding looking at the monitor screen for 5 minutes of the session – reactions not caused by somatic factors | 4 |
| The patient closes his/her eyes and/or turns his/her head avoiding looking at the monitor screen for 15 minutes of the session – reactions not caused by somatic factors | 3 |
| The patient closes his/her eyes and/or turns his/her head avoiding looking at the monitor screen for 25 minutes of the session – reactions not caused by somatic factors | 2 |
| The patient does not maintain his/her gaze on the monitor screen (closing the eyes, turning the head to avoid looking at the monitor screen, looking outside the monitor screen) – reactions not caused by somatic factors | 1 |
| **General activity (GA)** |  |
| The patient performs the tasks during the whole session + attempts to correct errors | 5 |
| The patient performs the tasks for 20 – 30 minutes | 4 |
| The patient performs the tasks during the period when he/she maintains his/her gaze on the screen and/or attempts to correct errors (less than 15 minutes) | 3 |
| The patient performs the tasks during the period when he/she maintains his/her gaze on the screen (less than 15 minutes) | 2 |
| The patient closes his/her eyes and is clearly unwilling to perform the tasks (before the session the patient was fully active) | 1 |

**Supplementary Table S5.** Assessment of the ability to maintain attention

| **Observation** | **Score** |
| --- | --- |
| The patient completed the task without taking a break during the whole session | Attention properly maintained (P) |
| The patient interrupted performing the task once or twice (not affected by somatic factors) | A light deficit in attention (L) |
| The patient interrupted performing the task at least three times (not affected by somatic factors) | A severe deficit in attention (V) |

**Supplementary Table S6.** Results of the assessment of the ability to read. P – ability preserved, L – light impairment, M – moderate impairment, V – severe impairment, C – ability ceased.

| **ID** | **Task no.** | | | | | | | | | **Score** | **Symbol** |
| --- | --- | --- | --- | --- | --- | --- | --- | --- | --- | --- | --- |
|  | **1** | **2** | **3** | **4** | **5** | **6** | **7** | **8** | **9** |  |  |
| 1 | 4 | 5 | 5 | 5 | 5 | 5 | 5 | 3 | 5 | **42** | **P** |
| 2 | 4 | 5 | 3 | 5 | 2 | 5 | 5 | 5 | 5 | **39** | **L** |
| 3 | 4 | 5 | 4 | 1 | 3 | 1 | 5 | 5 | 4 | **32** | **L** |
| 4 | 5 | 4 | 2 | 5 | 5 | 5 | 5 | 5 | 4 | **40** | **P** |
| 5 | 5 | 5 | 3 | 1 | 5 | 1 | 5 | 4 | 3 | **32** | **L** |
| 6 | 5 | 5 | 3 | 5 | 3 | 5 | 5 | 3 | 4 | **38** | **L** |
| 7 | 4 | 5 | 4 | 5 | 5 | 5 | 5 | 5 | 5 | **43** | **P** |
| 8 | 4 | 5 | 4 | 4 | 5 | 4 | 5 | 5 | 5 | **41** | **P** |
| 9 | 3 | 5 | 4 | 5 | 5 | 5 | 5 | 3 | 5 | **40** | **P** |
| 10 | 5 | 5 | 3 | 1 | 1 | 1 | 5 | 1 | 1 | **23** | **M** |
| 11 | 5 | 2 | 2 | 1 | 2 | 2 | 3 | 1 | 1 | **19** | **V** |
| 12 | 4 | 5 | 5 | 5 | 4 | 5 | 5 | 4 | 5 | **42** | **P** |
| 13 | 5 | 5 | 4 | 1 | 4 | 4 | 5 | 3 | 1 | **32** | **L** |
| 14 | 5 | 5 | 4 | 4 | 5 | 4 | 5 | 5 | 1 | **38** | **L** |
| 15 | 5 | 5 | 3 | 4 | 5 | 4 | 4 | 5 | 5 | **40** | **P** |
| 16 | 3 | 4 | 3 | 1 | 1 | 1 | 1 | 3 | 1 | **18** | **V** |
| 17 | 5 | 5 | 3 | 4 | 1 | 4 | 4 | 5 | 1 | **32** | **L** |
| 18 | 4 | 4 | 4 | 1 | 5 | 4 | 4 | 5 | 1 | **32** | **L** |
| 19 | 5 | 5 | 5 | 2 | 5 | 2 | 5 | 3 | 5 | **37** | **L** |
| 20 | 1 | 5 | 2 | 1 | 3 | 1 | 1 | 5 | 5 | **24** | **M** |
| 21 | 1 | 4 | 4 | 1 | 1 | 1 | 1 | 2 | 1 | **16** | **V** |
| 22 | 5 | 5 | 4 | 5 | 4 | 4 | 5 | 4 | 4 | **40** | **P** |
| 23 | 5 | 5 | 5 | 5 | 5 | 5 | 5 | 5 | 5 | **45** | **P** |
| 24 | 5 | 5 | 3 | 3 | 5 | 4 | 5 | 5 | 1 | **36** | **L** |
| 25 | 4 | 5 | 3 | 4 | 5 | 4 | 3 | 5 | 5 | **38** | **L** |
| 26 | 5 | 5 | 3 | 2 | 1 | 3 | 1 | 1 | 1 | **22** | **M** |
| 27 | 3 | 4 | 4 | 3 | 5 | 4 | 5 | 5 | 1 | **34** | **L** |
| 28 | 5 | 5 | 5 | 5 | 5 | 5 | 5 | 3 | 1 | **39** | **L** |
| 29 | 5 | 5 | 5 | 5 | 5 | 4 | 5 | 5 | 5 | **44** | **P** |
| 30 | 5 | 5 | 3 | 1 | 1 | 1 | 1 | 5 | 1 | **23** | **M** |
| 31 | 4 | 5 | 5 | 1 | 4 | 2 | 4 | 1 | 1 | **27** | **M** |
| 32 | 5 | 5 | 3 | 2 | 1 | 3 | 5 | 1 | 1 | **26** | **M** |
| 33 | 5 | 5 | 2 | 2 | 1 | 4 | 5 | 5 | 1 | **30** | **M** |
| 34 | 5 | 4 | 3 | 2 | 1 | 2 | 1 | 1 | 1 | **20** | **V** |
| 35 | 5 | 5 | 4 | 2 | 4 | 3 | 5 | 4 | 3 | **35** | **L** |
| 36 | 5 | 5 | 5 | 5 | 4 | 5 | 5 | 5 | 4 | **43** | **P** |
| 37 | 5 | 5 | 4 | 4 | 1 | 5 | 5 | 5 | 1 | **35** | **L** |
| 38 | 5 | 5 | 4 | 5 | 4 | 5 | 5 | 4 | 5 | **42** | **P** |
| 39 | 5 | 5 | 3 | 1 | 1 | 4 | 4 | 1 | 1 | **25** | **M** |
| 40 | 3 | 4 | 4 | 3 | 4 | 4 | 5 | 4 | 1 | **32** | **L** |
| 41 | 5 | 5 | 2 | 5 | 4 | 5 | 5 | 5 | 5 | **41** | **P** |
| 42 | 4 | 5 | 5 | 1 | 5 | 5 | 5 | 1 | 1 | **32** | **L** |
| 43 | 4 | 4 | 2 | 1 | 2 | 4 | 5 | 2 | 3 | **27** | **M** |
| 44 | 5 | 5 | 5 | 4 | 4 | 5 | 5 | 3 | 1 | **37** | **L** |
| 45 | 2 | 4 | 4 | 5 | 3 | 5 | 5 | 5 | 1 | **34** | **L** |
| 46 | 5 | 5 | 5 | 1 | 4 | 5 | 5 | 5 | 4 | **39** | **L** |
| 47 | 5 | 5 | 5 | 5 | 5 | 5 | 5 | 5 | 5 | **45** | **P** |
| 48 | 5 | 5 | 4 | 4 | 1 | 5 | 5 | 5 | 1 | **35** | **L** |
| 49 | 5 | 5 | 5 | 1 | 5 | 4 | 5 | 5 | 1 | **36** | **L** |
| 50 | 5 | 5 | 5 | 1 | 1 | 5 | 5 | 5 | 5 | **37** | **L** |

**Supplementary Table S7.** Results of the assessment of motivation. MC – muscle contraction, MG – maintaining gaze, GA – general activity. H – High, M – Moderate, A – Anti-motivated.

| **ID** | **Session I** | | | | **Session II** | | | | **Session III** | | | |
| --- | --- | --- | --- | --- | --- | --- | --- | --- | --- | --- | --- | --- |
|  | **MC** | **MG** | **GA** | **Sum** | **MC** | **MG** | **GA** | **Sum** | **MC** | **MG** | **GA** | **Sum** |
| 1 | 3 | 4 | 3 | **10 - H** | 4 | 4 | 3 | **11 - H** | 3 | 4 | 2 | **9 - M** |
| 2 | 5 | 4 | 3 | **12 - H** | 4 | 4 | 3 | **11 - H** | 4 | 5 | 3 | **12 - H** |
| 3 | 2 | 2 | 2 | **6 - M** | 2 | 3 | 2 | **7 - M** | 2 | 2 | 1 | **5 - M** |
| 4 | 4 | 5 | 5 | **14 - H** | 5 | 5 | 5 | **15 - H** | - | | | |
| 5 | 1 | 2 | 1 | **4 - A** | 1 | 2 | 1 | **4 - A** | 2 | 3 | 2 | **7 - M** |
| 6 | 3 | 3 | 2 | **8 - M** | 4 | 4 | 4 | **12 - H** | 5 | 5 | 5 | **15 - H** |
| 7 | 3 | 3 | 2 | **8 - M** | 3 | 3 | 3 | **9 - M** | 4 | 4 | 3 | **11 - H** |
| 8 | 4 | 5 | 5 | **14 - H** | 5 | 5 | 5 | **15 - H** | - | | | |
| 9 | 3 | 3 | 3 | **9 - M** | 3 | 3 | 3 | **9 - M** | 3 | 3 | 3 | **9 - M** |
| 10 | 3 | 3 | 3 | **9 - M** | 4 | 3 | 3 | **10 - H** | 4 | 3 | 3 | **10 - H** |
| 11 | 1 | 1 | 1 | **3 - A** | 1 | 2 | 2 | **5 - M** | 1 | 2 | 1 | **4 - A** |
| 12 | 3 | 3 | 2 | **8 - M** | 4 | 3 | 2 | **9 - M** | 4 | 3 | 3 | **10 - H** |
| 13 | 1 | 2 | 2 | **5 - M** | 2 | 2 | 2 | **6 - M** | 2 | 2 | 2 | **6 - M** |
| 14 | 4 | 3 | 4 | **11 - H** | 4 | 3 | 4 | **11 - H** | 5 | 4 | 5 | **14 - H** |
| 15 | 3 | 4 | 2 | **9 - M** | 4 | 4 | 3 | **11 - H** | 4 | 4 | 4 | **12 - H** |
| 16 | 1 | 2 | 1 | **4 - A** | 1 | 2 | 1 | **4 - A** | 1 | 2 | 1 | **4 - A** |
| 17 | 3 | 3 | 2 | **8 - M** | 3 | 3 | 2 | **8 - M** | 4 | 3 | 3 | **10 - H** |
| 18 | 3 | 3 | 2 | **8 - M** | 3 | 3 | 2 | **8 - M** | 3 | 3 | 2 | **8 - M** |
| 19 | 4 | 3 | 3 | **10 - H** | 4 | 3 | 3 | **10 - H** | 4 | 4 | 4 | **12 - H** |
| 20 | 2 | 3 | 2 | **7 - M** | 1 | 1 | 1 | **3 - A** | 1 | 2 | 2 | **5 - M** |
| 21 | 3 | 3 | 2 | **8 - M** | 3 | 3 | 3 | **9 - M** | 4 | 3 | 2 | **9 - M** |
| 22 | 4 | 4 | 4 | **12 - H** | 5 | 4 | 5 | **14 - H** | 5 | 5 | 5 | **15 - H** |
| 23 | 1 | 3 | 2 | **6 - M** | 1 | 3 | 2 | **6 - M** | 1 | 3 | 2 | **6 - M** |
| 24 | 3 | 3 | 2 | **8 - M** | 3 | 4 | 3 | **10 - H** | 4 | 4 | 2 | **10 - H** |
| 25 | 3 | 4 | 4 | **11 - H** | 4 | 5 | 5 | **14 - H** | 4 | 5 | 5 | **14 - H** |
| 26 | 1 | 2 | 2 | **5 - M** | 2 | 3 | 2 | **7 - M** | 2 | 3 | 2 | **7 - M** |
| 27 | 3 | 4 | 3 | **10 - H** | 4 | 4 | 3 | **11 - H** | 4 | 4 | 3 | **11 - H** |
| 28 | 4 | 4 | 2 | **10 - H** | 5 | 4 | 3 | **12 - H** | 4 | 5 | 4 | **13 - H** |
| 29 | 1 | 2 | 2 | **5 - M** | 1 | 2 | 1 | **4 - A** | 1 | 3 | 2 | **6 - M** |
| 30 | 1 | 3 | 2 | **6 - M** | 1 | 3 | 2 | **6 - M** | 1 | 3 | 2 | **6 - M** |
| 31 | 1 | 3 | 2 | **6 - M** | 2 | 3 | 2 | **7 - M** | 2 | 3 | 1 | **6 - M** |
| 32 | 2 | 3 | 2 | **7 - M** | 2 | 3 | 2 | **7 - M** | 3 | 4 | 2 | **9 - M** |
| 33 | 4 | 4 | 4 | **12 - H** | 4 | 4 | 3 | **11 - H** | 4 | 5 | 4 | **13 - H** |
| 34 | 2 | 3 | 2 | **7 - M** | 2 | 3 | 2 | **7 - M** | 2 | 3 | 2 | **7 - M** |
| 35 | 4 | 4 | 4 | **12 - H** | 4 | 3 | 2 | **9 - M** | 4 | 4 | 4 | **12 - H** |
| 36 | 4 | 5 | 5 | **14 - H** | 4 | 5 | 5 | **14- P** | 5 | 5 | 5 | **15 - H** |
| 37 | 5 | 4 | 2 | **11 - H** | 4 | 5 | 4 | **13 - H** | 5 | 5 | 4 | **14 - H** |
| 38 | 4 | 5 | 5 | **14 - H** | 4 | 5 | 5 | **14 - H** | 4 | 5 | 5 | **14 - H** |
| 39 | 4 | 4 | 4 | **12 - H** | 4 | 3 | 2 | **9 - M** | 4 | 4 | 4 | **12 - H** |
| 40 | 4 | 3 | 2 | **9 - M** | 4 | 3 | 2 | **9 - M** | 4 | 4 | 4 | **12 - H** |
| 41 | 2 | 3 | 2 | **7 - M** | 3 | 3 | 2 | **8 - M** | 2 | 3 | 2 | **7 - M** |
| 42 | 3 | 3 | 2 | **8 - M** | 3 | 4 | 4 | **11 - H** | 4 | 4 | 4 | **12 - H** |
| 43 | 2 | 3 | 2 | **7 - M** | 2 | 3 | 2 | **7 - M** | 2 | 3 | 2 | **7 - M** |
| 44 | 1 | 3 | 2 | **6 - M** | 1 | 3 | 2 | **6 - M** | 1 | 3 | 2 | **6 - M** |
| 45 | 4 | 4 | 4 | **12 - H** | 4 | 5 | 5 | **14 - H** | 4 | 5 | 5 | **14 - H** |
| 46 | 2 | 4 | 4 | **10 - H** | 4 | 4 | 4 | **12 - H** | 4 | 4 | 4 | **12 - H** |
| 47 | 4 | 5 | 5 | **14 - H** | 4 | 5 | 5 | **14 - H** | 5 | 5 | 4 | **14 - H** |
| 48 | 1 | 4 | 4 | **9 - M** | 3 | 4 | 4 | **11 - H** | 4 | 4 | 4 | **12 - H** |
| 49 | 3 | 3 | 2 | **8 - M** | 3 | 4 | 4 | **11 - H** | 3 | 3 | 2 | **8 - M** |
| 50 | 5 | 4 | 4 | **13 - H** | 5 | 3 | 3 | **11 - H** | 5 | 4 | 4 | **13 - H** |

**Supplementary Table S8.** Results of the assessment of the ability to maintain attention. P – attention properly maintained, L – light deficit, V – severe deficit.

| **ID** | **Session I** | **Session II** | **Session III** |
| --- | --- | --- | --- |
| 1 | P | L | P |
| 2 | P | P | P |
| 3 | L | L | L |
| 4 | V | L | - |
| 5 | V | V | L |
| 6 | P | P | P |
| 7 | V | V | V |
| 8 | P | P | - |
| 9 | L | V | L |
| 10 | L | V | L |
| 11 | V | V | V |
| 12 | P | P | P |
| 13 | L | L | L |
| 14 | P | P | P |
| 15 | P | P | P |
| 16 | V | V | V |
| 17 | L | L | L |
| 18 | L | P | L |
| 19 | V | V | V |
| 20 | L | V | V |
| 21 | P | P | P |
| 22 | P | P | P |
| 23 | L | L | L |
| 24 | V | V | V |
| 25 | P | P | P |
| 26 | V | L | L |
| 27 | P | P | L |
| 28 | P | P | P |
| 29 | V | V | V |
| 30 | L | V | V |
| 31 | L | L | V |
| 32 | V | V | V |
| 33 | L | L | P |
| 34 | L | L | L |
| 35 | P | P | P |
| 36 | L | V | L |
| 37 | L | P | L |
| 38 | P | P | P |
| 39 | L | V | L |
| 40 | L | V | L |
| 41 | V | V | V |
| 42 | L | L | P |
| 43 | L | L | L |
| 44 | L | L | V |
| 45 | L | P | P |
| 46 | V | V | L |
| 47 | P | P | P |
| 48 | L | P | P |
| 49 | V | L | L |
| 50 | P | L | L |
